# Overcoming the diagnostic gap in mild cognitive impairment in Parkinson’s disease: a pilot study employing a machine learning-/augmented reality-based digital biomarker

**DOI:** 10.3389/fnagi.2026.1839000

**Published:** 2026-06-01

**Authors:** Karolina Poplawska-Domaszewicz, Emmanuel Streel, Aleksandra Brek, Natalia Miśko, Ewa Kosińska, Vinod Metta, Per Odin, Angelo Antonini, Roberta Biundo, Eleonora Fiorenzato, Kit Wu, Saivansh Chopra, Srikaanth Haridas, Ioannis Tarnanas, Nicholas Griffin, Victoria Brugada-Ramentol, M Florencia Iulita, Madison Jones, Slawomir Michalak, Wojciech Kozubski, Kallol Ray Chaudhuri

**Affiliations:** 1 Institute of Neurological Disorders, Poznan University of Medical Sciences, Poznań, Poland; 2 Altoida Inc., Washington, DC, United States; 3 University Hospital, Poznan, Poland; 4 Kings Parkinson’s Centre of Excellence, King’s College Hospital, Dubai Hills, Dubai, United Arab Emirates; 5 Kings College Hospital, London, United Kingdom; 6 Department of Neurology, Lund University, Lund, Sweden; 7 Neurodegenerative Disease Unit, Department of Neuroscience, Padua Neuroscience Center (PNC), University of Padua, Padua, Italy; 8 IRCCS San Camillo, Venice, Italy; 9 Department of General Psychology, University of Padova, Padua, Italy; 10 Dementech Neuroscience Clinical Academic Centre, London, United Kingdom

**Keywords:** cholinergic, cognition, digital biomarkers, mild cognitive impairment, Parkinson’s disease

## Abstract

**Background:**

Cognitive impairment is a clinically significant, non-motor symptom of Parkinson’s disease (PD) commonly associated with reduced quality of life, increased caregiver burden, and higher risk of progression to dementia. Mild cognitive impairment in PD (PD-MCI) is expressed heterogeneously, with likely prognostic implications. This pilot study evaluated the feasibility and preliminary diagnostic performance of a Machine Learning/Augmented Reality (ML/AR)-based digital assessment for identifying PD-MCI to compare with clinician-led classification.

**Methods:**

The Altoida NeuroMarker (hereafter, “NeuroMarker”) is a 10 min, self-administered digital cognitive and functional assessment performed by tablet, comprised of thirteen task challenges. The NeuroMarker was administered to 21 patients with PD. NeuroMarker-based MCI classification was compared to clinician-led classification using a confusion matrix to compute sensitivity, specificity, PPV, NPV, accuracy, and Cohen’s κ. Clinical assessments included the MMSE, ACE-III, Hoehn and Yahr stage, and BDI.

**Results:**

The NeuroMarker identified all six clinician-classified PD-MCI cases and classified an additional 11 patients with likely MCI. Sensitivity was 100% (95% CI: 54.1–100), specificity was 26.7% (95% CI: 7.8–55.1), PPV was 35.3% (95% CI: 14.2–61.7), NPV was 100% (95% CI: 39.8–100), accuracy was 47.6% (95% CI: 25.7–70.2), and κ = 0.17. Group differences were observed for age, ACE-III, sex, and education.

**Conclusion:**

These preliminary findings suggest that the NeuroMarker may identify clinician-recognized PD-MCI cases, with the potential to also flag patients with early or subthreshold cognitive impairment. However, the study’s wide confidence intervals, low agreement, smaller sample size, and absence of longitudinal confirmation limit interpretation. Larger studies utilizing comprehensive neuropsychological assessment and longitudinal follow-up are required.

## Introduction

Cognitive impairment is one of the most prevalent and clinically significant non-motor symptoms of Parkinson’s disease (PD), associated with reduced patient quality of life, increased caregiver strain, greater healthcare utilization, more severe and intrusive depressive symptoms, and impaired instrumental daily functioning (Bezdicek et al., 2025; Biundo et al., 2025; Poewe et al., 2017). Mild cognitive impairment in PD (PD-MCI) is a particularly integral consideration for clinicians, as a transitional state often preceding full-blown Parkinson’s disease dementia (PDD) (Biundo et al., 2025). The prodromal state of PD is characterized by an at risk period, a preclinical period and finally a prodromal period where clinical signs emerge and MCI features as one of the prodromal symptoms.

Accordingly, cognitive dysfunction, and PD-MCI in particular, has become a major focus of contemporary Parkinson’s disease research (Huang et al., 2025). Given the emergence of MCI in the disease’s prodromal stage, both disease-modifying and immunomodulatory treatment strategies are being researched to slow dementia progression. Recent advances in clinical phenotyping may require more subtype-specific and personalized therapeutic approaches (Marras et al., 2020; Stocchi et al., 2024).

As an example, the proposed cholinergic subtype of Parkinson’s disease (PD) is expressed during the more transitory stages of MCI-PD (Aarsland et al., 2021). More recent subtyping of Parkinson’s disease with mild cognitive impairment using the Cholinergic nucleus 4 (Ch4) volume has also been proposed so as to enrich clinical trial populations and scale to develop personalized treatments (Negida et al., 2025). Studies have shown that 19–62% of patients with PD-MCI may develop dementia within 2–5 years of diagnosis, underscoring the importance of early detection (Wood et al., 2016). Studies on the Campaign cohort from Cambridge in the UK, in addition to findings from a 5-year cohort study from the Norwegian Movement Disorder Center, reported a conversion rate to PDD of 39–50% among patients with PD-MCI (Greenland et al., 2019; Pedersen et al., 2017; Williams-Gray et al., 2009). Furthermore, annual progression rates to dementia in PD-MCI patients have been estimated at 6–15% (Monastero et al., 2018). Notably, PD-MCI is an independent risk factor for the development of PDD and carries prognostic significance even as cognitive function appears to stabilize or improve (Baiano et al., 2020; Greenland et al., 2019). Cognitive tests have been proposed as an integral part of the Chaudhuri dashboard for PD (Qamar et al., 2023).

Diagnosing PD-MCI remains a challenge due to its subtle and heterogeneous presentation and system-wide reliance on clinical scales and patient interviews for detection. The latest MDS reviews by Bezdicek et al. (2025) and Biundo et al. (2025) highlight the ongoing need for improved cognitive assessment in PD. Despite the ready-availability of various tools evaluating memory, language, and visuospatial function, many lack sufficient sensitivity to detect treatment effects, correlate poorly with cognitive biomarkers, and remain underdeveloped in terms of digital transformation (Campagnolo et al., 2025). Further validation of both existing and novel instruments is essential in advancing cognitive diagnostics in PD and to support the field of translational neuroscience (Bezdicek et al., 2025; Biundo et al., 2025). Accurate PD-MCI detection will be especially vital in genetic, early-onset PD as bespoke genomic personalized therapies become available in future, including those with glucocerebrosidase mutations (GBA 1) (Poplawska-Domaszewicz et al., 2024).

The digital cognitive assessment field is active and evolving, with several tools already having been leveraged in neurodegenerative disorder detection, including the CANTAB, Mezurio, and DailyCog assessment batteries. These approaches have advanced computerized and remote cognitive testing, including in Parkinson’s disease and other dementia-risk populations. However, the NeuroMarker platform differs from many conventional computerized cognitive batteries in that it joins functional, AR-based tasks with motoric, speech/language, and task-performance signals, which are then processed using a pre-existing machine learning algorithm to generate an MCI classification. Though digital cognitive assessments are not novel *per se*, an AR-based, multimodal functional assessment that could capture cognitive, motor, and daily function has not yet been realized in the diagnostic framework. There is further interest in its ability to capture additional, relevant features not yet represented in full by traditional paper- or screen-based cognitive tests.

Currently, there is growing interest in the use of artificial intelligence (AI) as an adjunctive diagnostic tool to support holistic care in Parkinson’s disease. AI-based approaches may enhance the early identification and monitoring of cognitive decline, thereby improving patient outcomes through more personalized management strategies (Bezdicek et al., 2025; Huang et al., 2025). To address this unmet need, we conducted a proof-of-concept study in 21 patients with advanced Parkinson’s disease using the NeuroMarker. Through a series of motoric, augmented reality, and language tasks, the platform allows for a very sensitive identification of cognitive anomalies in 10–12 min. Previous independent research in 121 subjects with the RADAR-AD consortium found that the platform allowed for the identification of cognitive anomalies in preclinical Alzheimer’s, unlike traditional cognitive assessments (Muurling et al., 2023). Prior validation of this approach has primarily been conducted in Alzheimer’s disease populations. Its application to Parkinson’s disease, where motor impairment may influence performance on digital tasks, remains to be established and warrants specific evaluation.

Given the high prevalence and prognostic importance of PD-MCI, coupled with the limitations of current clinical assessments, there is a clear need for sensitive and scalable approaches to improve early detection (Campagnolo et al., 2025). Digital biomarkers, particularly those integrating artificial intelligence and augmented reality, offer the potential to capture subtle cognitive deficits that may be overlooked in routine clinical practice. Building on prior evidence of the NeuroMarker platform in Alzheimer’s disease, we aimed in this pilot study to evaluate its feasibility and diagnostic performance in patients with Parkinson’s disease. Specifically, we sought to compare NeuroMarker-based classification of PD-MCI with clinician-led diagnosis and to explore demographic and cognitive correlates of classification outcomes (Poplawska-Domaszewicz et al., 2025). If validated in larger cohorts, such tools could have broad implications for the field, supporting earlier screening, enabling longitudinal monitoring, and informing more personalized treatment strategies in Parkinson’s disease.

## Materials and methods

### Study design and participants

Patients diagnosed with idiopathic Parkinson’s disease (PD), as defined by the Movement Disorders Society Criteria and attending the Movement Disorders Clinic, were recruited to the study (Postuma et al., 2015). Patients were then clinically assessed for the presence or absence of MCI using the Movement Disorders Society Task Force Criteria, utilizing the Level 1 (abbreviated) criteria, and the Mini-Mental State Examination (MMSE).

The inclusion criteria are described below:

Inclusion criteria: Age ≥ 18.

1. Clinical diagnosis of PD per MDS criteria.
2. Stable PD medications for ≥ 30 days prior.
3. Ability to provide informed consent.

Exclusion criteria:

1. Hoehn and Yahr (H&Y) stage V and institutionalized.
2. Pregnant or nursing patients.
3. Conditions interfering with assessment (e.g., dementia, Mini-Mental State Examination (MMSE) < 21).
4. Inability to understand the cognitive battery

### Digital biomarker

Participants completed the NeuroMarker assessment at the study center in a self-administered manner using iOS tablets. Before starting the assessment, study personnel provided a brief explanation of the purpose of the test and the overall procedure. Participants then completed a brief in-app tutorial to familiarize themselves with the assessment interface and task format. At the domain level, the thirteen NeuroMarker challenges include shape-drawing, tapping, balancing, augmented-reality, and speech/language tasks. Together, these tasks are designed to capture motoric, visuospatial, executive, memory, and language-related performance features. Details of the assessment structure have been previously described by Muurling et al. (2023). with additional detailed information about the model in Buegler et al. (2020) and Tarnanas et al. (2015).

The NeuroMarker platform applies a pre-existing, proprietary machine learning algorithm to map multimodal, task-derived signals and generate a binary classification that indicates the presence or absence of MCI. The present study did not involve development, retraining, or recalibration of the algorithm for Parkinson’s disease, and therefore evaluates the feasibility and preliminary diagnostic performance of the existing algorithm in a PD population.

### Comparative standard measures

The clinical reference classification was based on clinician-led assessment of the presence or absence of PD-MCI, supported by available cognitive and clinical measures, including the Mini-Mental State Examination (MMSE), Addenbrooke’s Cognitive Examination III (ACE-III), the Hoehn-Yahr stage, and the Beck Depression Inventory (BDI). The ACE-III was administered by the clinical team in the region’s appropriate local language.

A full MDS Level II neuropsychological assessment was not performed. Therefore, the clinician-led classification should be interpreted as a clinical reference standard rather than a definitive gold-standard diagnosis of PD-MCI. Clinician-led PD-MCI classification was performed with the NeuroMarker output blinded. NeuroMarker classification was generated independently and was not released to the clinicians at the time of clinical assessment.

### Ethical approval

The study was approved by the ethical committee of Poznan University of Medical Sciences (414/25).

### Statistical analysis

Continuous variables were summarized as mean ± SD and categorical variables as counts (%). Agreement between clinician- and NeuroMarker-based MCI classification was assessed using a 2 × 2 confusion matrix from which sensitivity and specificity were computed; inter-method agreement was quantified with Cohen’s κ. For group comparisons across the three strata (A: MCI by both; B: MCI by NeuroMarker only; C: no MCI by either), continuous variables were analyzed with the Kruskal-Wallis test, and categorical variables with chi-square. When the Kruskal-Wallis test was significant (*p* < 0.05), pairwise tests were performed with Bonferroni correction (three contrasts; adjusted α = 0.0167); adjusted *p*-values are reported for pairwise results. All tests were two-sided. Analyses were conducted in IBM SPSS Statistics v29.0.2.0. Agreement was summarized in a confusion matrix; sensitivity, specificity, PPV, NPV, accuracy, and Cohen’s κ were computed. Participants were grouped as: A (MCI by both), B (MCI by NeuroMarker only), C (no MCI by either).

Owing to the exploratory nature of this study, which focused on evaluating the feasibility and preliminary diagnostic performance of a device-based assessment rather than testing confirmatory hypotheses, a formal sample size calculation was not performed. Continuous variables were compared to Kruska-Wallis; categorical with chi-square; significance p < 0.05. Bonferroni correction (α = 0.0167) was applied to *post-hoc* pairwise tests. Analyses used IBM SPSS Statistics v29.0.2.0.

## Results

Data from 21 individuals with Parkinson’s disease were analyzed for age, sex, education, ACE-III, MMSE, Hoehn and Yahr stage, and BDI. Baseline characteristics of the full cohort (*n* = 21) are summarized in Table 1.

**TABLE 1:** Population description.

Age	Sex	Level_education	HoehnYahr	ACE-III	MMSE	Beck
63	Male	High school diploma	3	86	28	20
60	Male	High school diploma	3	87	25	16
71	Female	High school diploma	3	81	26	6
70	Female	High school diploma	3	84	28	18
79	Female	College or university degree	4	78	26	23
75	Female	College or university degree	3	77	26	33
71	Male	No high school	3	70	23	21
64	Male	No high school	4	80	19	2
71	Male	No high school	3	67	22	20
38	Male	College or university degree	2	94	29	14
53	Male	No high school	1	97	28	16
76	Male	High school diploma	3	89	28	12
70	Female	College or university degree	3	89	28	25
66	Male	College or university degree	3	96	29	2
60	Male	High school diploma	2	81	25	4
54	Male	College or university degree	3	91	29	5
65	Female	College or university degree	3	95	30	7
44	Male	College or university degree	3	99	29	4
53	Male	College or university degree	2	98	28	4
56	Male	College or University degree	3	99	30	5
39	Male	College or university degree	3	99	29	7

Values are presented as individual participant data. ACE-III = Addenbrooke’s Cognitive Examination III; MMSE = Mini-Mental State Examination; Beck = Beck Depression Inventory; Hoehn and Yahr = Parkinson’s disease staging scale.

Each participant was classified as MCI/non-MCI by a clinician and by a digital cognitive biomarker (NeuroMarker).

The NeuroMarker identified all six clinician-classified MCI cases plus 11 additional cases (Table 2), corresponding to a sensitivity of 100% (95% CI: 54.1–100), a specificity of 26.7% (95% CI: 7.8–55.1), PPV of 35.3% (95% CI: 14.2–61.7), NPV of 100% (95% CI: 39.8–100), accuracy of 47.6% (95% CI: 25.7–70.2), and κ = 0.17. Overall group differences were observed for age (*p* = 0.024), ACE-III (*p* = 0.005), and for sex (*p* = 0.040) and education (*p* = 0.033); MMSE (*p* = 0.095) and BDI (*p* = 0.052) were not significant. Characteristics of groups A, B, and C are shown in Table 3. After Bonferroni-adjusted pairwise testing, ACE-III differed between Groups A and C (unadjusted *p* = 0.004; Bonferroni-adjusted *p* = 0.012) (Table 4).

**TABLE 2:** Confusion matrix and performance metrics for NeuroMarker and clinician classification of MCI.

	NeuroMarker MCI (+)	NeuroMarker MCI (–)
Clinician MCI (+)	6	0
Clinician MCI (–)	11	4

NeuroMarker identified all six clinician-classified MCI. MCI, mild cognitive impairment; PPV, positive predictive value; NPV, negative predictive value. Sensitivity: 100% (95% CI: 54.1–100) Specificity: 26.7% (95% CI: 7.8–55.1) PPV: 35.3% (95% CI: 14.2–61.7) NPV: 100% (95% CI: 39.8–100) Accuracy: 47.6% (95% CI: 25.7–70.2).

**TABLE 3:** Characteristics of the three groups.

Group	n	Age	ACE-III	MMSE	Hoehn and Yahr	Beck
A—MCI by both	6	69.7 ± 7.1	82.2 ± 4.2	24.3 ± 2.3	3.17 ± 0.41	19.3 ± 8.85
B—MCI by NeuroMarker only	11	62.5 ± 10.8	86.3 ± 10.4	27.5 ± 1.7	2.64 ± 0.84	11.6 ± 8.19
C—No MCI by either	4	48.0 ± 7.9	98.8 ± 0.50	29.3 ± 0.96	2.50 ± 0.58	5.0 ± 1.41

Group A: MCI detected by NeuroMarker and clinical testing (mean ± SD), Group B: MCI by NeuroMarker only and Group C: No MCI by either, MCI, Mild cognitive impairment; Beck, Beck depression inventory, MMSE + Mini-mental state examination; ACE, Addenbrooke’s cognitive examination.

**TABLE 4:** Pairwise comparisons of all variables.

Variable	Comparison	p-unadjusted	Result*
Age	A vs. B	0.724	NS
Age	A vs. C	0.020	NS
Age	B vs. C	0.14	NS
ACE-III	A vs. B	0.806	NS
ACE-III	A vs. C	0.004	Significant
ACE-III	B vs. C	0.031	NS
MMSE	A vs. B	0.416	NS
MMSE	A vs. C	0.032	NS
MMSE	B vs. C	0.096	NS
Beck	A vs. B	0.306	NS
Beck	A vs. C	0.052	NS
Beck	B vs. C	0.306	NS
Hoehn–Yahr	A vs. B	0.235	NS
Hoehn–Yahr	A vs. C	0.224	NS
Hoehn–Yahr	B vs. C	1.000	NS

ACE, Addenbrooke’s cognitive examination; NS, Not significant. *Unadjusted *p*-values are reported; statistical significance was determined using Bonferroni correction (α = 0.0167).

Group B (MCI by NeuroMarker only) indicated intermediate performance on ACE-III and MMSE (A < B < C; Table 3: ACE-III means 82.2, 86.3, 98.8; MMSE means 24.3, 27.5, 29.3). After Bonferroni correction, no pairwise differences involving Group B were significant (all p_adj ≥ 0.060). The only significant pairwise significant contrast was ACE-III: Group A vs. Group C (p_adj = 0.012; Table 4). Hoehn and Yahr stage did not differ significantly across groups in overall comparison (Kruskal–Wallis p = 0.36) or in pairwise comparisons after Bonferroni correction (all p_adj > 0.05).

## Discussion

Our data indicated that the NeuroMarker accurately identified all six cases of Parkinson’s disease (PD) that were clinically recognized as having MCI. In addition, the platform identified a further 11 cases not classified as MCI by clinicians (Table 2), yielding a sensitivity of 100% and a specificity of 26.7% (κ = 0.17). These estimates should be interpreted with caution due to the smaller sample size. In particular, the 100% sensitivity estimate is based on only six clinician-classified PD-MCI cases and has a wide confidence interval. The present findings therefore support feasibility and preliminary signal detection rather than definitive diagnostic accuracy.

Between-group analyses revealed significant differences for age (*p* = 0.024), ACE-III scores (*p* = 0.005), sex (*p* = 0.040), and education (*p* = 0.033), while the MMSE (*p* = 0.095) and BDI (*p* = 0.052) were not significant. After Bonferroni-adjusted pairwise testing, ACE-III scores differed significantly between Groups A and C (p_adj = 0.012).

The ACE-III pattern followed an ordered distribution across groups, with the lowest scores in patients classified as MCI by both clinical and NeuroMarker assessment (Group A). This ordered pattern suggests a gradient of cognitive performance across groups, consistent with increasing severity of cognitive impairment from Group C to Group A.

The key point of interest from our data suggests that while the NeuroMarker accurately identified all six cases of PD that were clinically considered to have MCI, a further 11 additional cases were considered to have MCI under NeuroMarker assessment (Table 1). These cases were not clinically identified as MCI, and accordingly despite a sensitivity of 100%, the assessment’s specificity was set at 26.7% (κ = 0.17).

In view of these results, our pilot data study suggests that the addition of the NeuroMarker to standard clinical assessment measures may enhance the sensitivity of MCI detection in Parkinson’s disease. Whether these additionally identified cases represent subtle cognitive impairment missed at clinical assessment or false-positive classifications cannot be determined from the present design. Longitudinal follow-up against a Level II reference standard is required.

it is likely the additionally-identified cases are from study participants with subtle or early cognitive impairment not clinically recognized at the time of assessment. Importantly, no clinician-identified MCI cases were missed by the platform. These findings warrant further evaluation in larger cohorts with longitudinal follow-up to clarify the clinical significance of these additional detections.

The data was also analyzed for between group differences and significant changes were observed for age (*p* = 0.024), for ACE-III (*p* = 0.005), and for sex (*p* = 0.040) and education (*p* = 0.033); MMSE (*p* = 0.095), and BDI (*p* = 0.052) comparisons were not significant.

Sex and education were heavily associated with MCI classification in this cohort. The NeuroMarker appeared to identify MCI more frequently among female participants, consistent with some prior reports of sex-related differences in cognitive trajectories in Parkinson’s disease (Cholerton et al., 2018). Similarly, education level was associated with classification outcome, potentially reflecting the influence of cognitive reserve on test performance. Given the small sample size and exploratory nature of these findings, these associations should be interpreted with caution; however, they suggest that demographic variables like sex and education may modulate both clinical and digital detection of PD-MCI and warrant systematic investigation in larger studies.

A difference in ACE-III scores was observed between Groups A and C; however, this comparison involves very small group sizes (*n* = 6 vs. *n* = 4) and should be interpreted with caution. This finding is exploratory and does not provide confirmatory evidence of validity. Importantly, the NeuroMarker-only group (Group B) exhibited intermediate ACE-III values, falling between the MCI-confirmed (Group A) and cognitively intact (Group C) groups (A < B < C in ACE-III performance). Although these differences did not reach statistical significance after correction, this consistent intermediate pattern suggests that the NeuroMarker may be sensitive to subtle cognitive changes that do not yet meet conventional clinical thresholds for PD-MCI.

The ACE-III gradient across Groups A, B, and C is compatible with detection of earlier impairment but also with motor- or demographic-driven misclassification at this sample size, and should be treated as hypothesis-generating.

This finding would need to be confirmed in a larger study. These findings also indicate that digital tools are able to complement traditional scales by identifying early cognitive vulnerability to allow for tailored monitoring and intervention strategies. Motor severity is an important potential confound in digital assessment application for Parkinson’s disease. Although the NeuroMarker includes motoric task components, Hoehn and Yahr stage did not differ significantly across groups in this cohort. However, given the small sample size, the absence of statistical significance does not exclude a potential influence of motor impairment on classification. Future studies should include larger samples and analyses explicitly adjusted for motor severity.

Patient experiences with tolerability of device use was also deemed acceptable. Despite language differences, patients found the thirteen tablet challenges acceptable and reasonable to use. This indication establishes the basis of a larger, subsequent cross-cultural study.

## Conclusion

Variants of MCI have now emerged as likely predictors of the cholinergic subtype of PD, where indications of more rapid progression to dementia have been observed (Aarsland et al., 2021; Williams-Gray et al., 2009). Early recognition and further analysis of detected cases of MCI may assist in identifying specific MCI variants like semantic difficulty, which may be predictors of worse cognitive outcome. This methodology is also integral to the recently-proposed stepped care approach to Parkinson’s disease, wherein the acts of copying intersection pentagons and lexical fluency are proposed as tests to pick up subtle forms of MCI (Williams-Gray et al., 2009). If validated in larger, longitudinal cohorts utilizing comprehensive neuropsychological assessment, as a screening tool, this approach could support earlier identification of cognitive vulnerability and more structured monitoring of cognitive change in Parkinson’s disease. Whether earlier identification of cognitive vulnerability in this population would translate into actionable management decisions can only be addressed by longitudinal studies with comprehensive neuropsychological assessment.

This study’s limitations include small sample size and translation use in platform directions, though translation has been utilized in other studies as well. We envisage a larger international, cross-cultural study to address these limitations and further validate the technology for widespread clinical use. While the observed low specificity could be considered a limitation, this result should be interpreted in the context of the reference standard used. Specificity was calculated relative to clinician judgment, as opposed to an independent, biomarker-based gold standard, and therefore may reflect differences in sensitivity between methods rather than true misclassification.

The NeuroMarker algorithm was not specifically trained or adapted for Parkinson’s disease. Given that motor impairment may influence performance on certain task components, the extent to which classification reflects cognitive versus motor features cannot be fully determined in this study. Disease-specific model development and validation will be required in future work. Because the algorithm was not specifically retrained or recalibrated for Parkinson’s disease, the findings should be interpreted as a preliminary evaluation of an existing digital biomarker in a PD cohort. Future studies should examine whether PD-specific model development improves classification performance and helps disentangle cognitive from motor contributions to task performance.

This proof-of-concept study demonstrates that a self-administered, tablet-based digital assessment can identify all clinically recognized cases of PD-MCI while additionally detecting a subset of patients with intermediate cognitive performance who may represent earlier or subthreshold cognitive impairment. These findings support the potential value of combining clinical judgment with sensitive digital biomarkers to improve early detection and monitoring of cognitive decline in Parkinson’s disease.

## Data Availability

The raw data supporting the conclusions of this article will be made available by the authors, without undue reservation.
